# Genetic Parameters for Novel Feedlot Profitability‐Related Traits in Nelore Cattle

**DOI:** 10.1111/jbg.70031

**Published:** 2025-11-19

**Authors:** Letícia Pereira, Fernando Baldi, Guilherme Jordão Magalhães Rosa, José Bento Sterman Ferraz, Tiago Zanett Albertini, Minos Carvalho, Raysildo Barbosa Lobo, Eduardo da Costa Eifert, Elisa Peripolli, Cláudio Ulhôa Magnabosco

**Affiliations:** ^1^ Department of Animal Science Federal University of Goiás Goiânia Brazil; ^2^ Department of Animal Science São Paulo State University—Júlio de Mesquita Filho (UNESP), Access way Prof. Paulo Donato Castelane Jaboticabal Brazil; ^3^ Department of Animal Science University of Wisconsin‐Madison Madison Wisconsin USA; ^4^ Department of Animal Science, Faculty of Animal Science and Food Engineering University of São Paulo Pirassununga São Paulo Brazil; ^5^ @Tech—Innovation Technology for Agriculture Piracicaba Brazil; ^6^ National Association of Breeders and Researchers (ANCP) Ribeirão Preto Brazil; ^7^ Embrapa Cerrados Planaltina Brazil

**Keywords:** genetic parameters, livestock precision, phenotyping, profit phenotype, zebu cattle

## Abstract

This study aimed to estimate (co)variance components and genetic parameters for accumulated profitability (AFP) and profit per kilogram of liveweight gain (PFT), and their relationships with weight at 450 days of age (W450), scrotal circumference at 365 days of age (SC365), age at first calving (AFC), probability of precocious calving at 30 months of age (PPC30), age at puberty in males (APM), stayability (STAY), accumulated cow productivity (ACP), rib eye area (REA), rump fat thickness (RFT), residual feed intake (RFI), dry‐matter intake (DMI), residual live weight gain (RG), and frame score (FRAME). Data of profitability from 3614 Nelore ( *Bos indicus* ) animals were used. The (co)variance components and genetic parameters were estimated using Bayesian inference in a multi‐trait animal model. The heritability estimates for AFP and PFT were 0.18 and 0.02, respectively. AFP and PFT exhibited moderate to high genetic correlations with growth traits (0.64 to 0.65), carcass (0.43 to 0.44), feed efficiency (0.72 to 0.88), and frame (0.44 to 0.77), except for the correlation with RFT (−0.10 to −0.68), RFI (−0.23 to 0.28), and between PFT and DMI (0.26). Low to high genetic correlations (−0.47 to 0.76) with female reproductive traits and low correlations (−0.03 to −0.21) with male reproductive traits were observed for both traits. These results provide important information for improving economic performance by including complementary AFP and PFT tools in the selection criteria. Such traits can be strategic tools for producers when identifying animals with greater genetic potential for profitability, supporting decision‐making in genetic planning and herd management.

## 1 Introduction

Factors such as management, global trade, concerns about animal health and welfare, and consumer preferences (Tonsor and Schroeder 2006), added to the ever‐decreasing reduction of productive land and the pressures imposed by population growth (Greenwood 2021) are challenges commonly cited in the livestock sector. As resources become scarcer and concerns related to environmental issues increase, efforts to increase efficiency in the production of red protein through genetic improvement and precision livestock farming have been increasingly addressed (Reynolds et al. 2010; Scollan et al. 2010). In this context, developing genetic improvement technologies becomes a considerable strategy that can contribute to more efficient, economically sustainable beef production with a smaller environmental footprint.

Conventional beef production systems generally include the cow‐calf system phase carried out on pasture and the rearing and finishing period that can occur in more intensive pastures or feedlots systems (Greenwood 2021). The finishing phase in the feedlot is a strategy that aims to intensify the production of kilograms of meat per unit area (Pacheco et al. 2014). However, some of these difficulties can be highlighted as costs related to nutritional input, price and demand variations, particularly genetic variability. Obtaining profit margins in feedlot has been a major challenge, as margins are often narrow and require economies of scale to ensure sustainable economic returns.

The adoption of new traits measured directly at finishing can help identify and select animals that result in higher performance and profitability. Traits related to (re)production are important for genetic improvement programs, as are new traits (Knap 2020), since most economically important traits are measured until the yearling stage, limiting the ability to obtain accurate phenotypes on animal productivity at this stage (Pereira et al. 2025). The use of new traits in finishing is essential to identify and select more efficient animals, since this phase represents the peak of the animal's performance (Pereira et al. 2025), that is, the maximum expression of important traits for slaughter and commercialization. In this context, studying accumulated profitability (AFP) and profit per kilogram of live weight gain (PFT) measured in feedlots becomes relevant as new auxiliary traits for meat producers.

AFP measures the total economic return of the animal during the confined period, while PFT measures the bioeconomic efficiency of the animal in producing more kilos of meat in less time in feedlot. Additionally, Pereira et al. 2025, through genome‐wide selection studies, characterised the genetic architecture of these two new traits and reported potential candidate genes related to feed efficiency, ingestive behaviour, carcass, fertility and growth traits, and biological processes for AFP and PFT. Incorporating these traits allows for early decision‐making, such as identifying animals with greater genetic merit for economic return in the initial growth phase of cow‐calf operations and minimising investment in nutritional inputs. For example, different strategies can be used for animals with lower economic and productive potential, increasing the economic margin and minimising the environmental impact at this stage.

Measuring phenotypes in feedlots results in opportunities to develop new tools and traits that can effectively contribute to increasing productivity while minimising the information window between the different production phases. Additionally, this information provides a better understanding of how selection criteria commonly used in genetic improvement programs affect the performance of animals in finishing. Thus, this study aimed to estimate the components of (co)variance and genetic parameters for new traits related to feedlot profitability and their genetic and residual correlations with growth, reproduction, carcass, feed efficiency, and body composition traits in Nellore cattle.

## 2 Materials and Methods

This study was exempt from evaluation by the Animal Ethics Committee (CEUA), as established by Law No. 11,794 of 08/10/2008 and Normative Resolution No. 51 of 05/19/2021 from the National Council for Animal Experimentation Control (CONCEA) because the data were obtained from an existing database.

### 2.1 Dataset

The dataset used in this study, related to growth, reproduction, carcass, and feed efficiency, originates from 27 herds participating in the Nelore Brazil Improvement Program, coordinated by the National Association of Breeders and Researchers (ANCP, Ribeirão Preto, São Paulo, Brazil) located in four Brazilian geographical regions (Midwest, Southeast, Northeast, and North). Data regarding accumulated profitability and profit per kilogram of liveweight gain were provided by the company @tech (https://techagr.com/beeftrader, Piracicaba, São Paulo, Brazil). The relationship matrix contained 116,815 animals, including 4248 sires and 49,106 dams. The animals that constituted the database had an average inbreeding coefficient of 1.11% and a proportion of 1.75% of inbred individuals in the total population.

A total of 2127 out of 3614 animals with AFP and PFT phenotypes were genotyped using the low‐density panel. (Clarifide Nelore 3.0). The genotype quality control (QC) excluded animals and SNPs from the dataset with call rates < 0.90. Additionally, SNPs with a minor allele frequency (MAF) < 0.05, Mendelian conflicts > 1%, monomorphic SNPs with redundant positions, SNPs deviating from Hardy–Weinberg equilibrium expectations, and those located on non‐autosomal chromosomes were also excluded. After QC, 2127 genotyped animals and 35,658 SNPs remained in the database for analysis.

### 2.2 Traits

#### 2.2.1 Growth

A growth trait considered in this study was adjusted weight at 450 days of age (W450, kg). The calculation of standardised weight was conducted through linear regression, considering the average daily gain assessed between 405 and 495 days of age for the variable W450 (Negreiros et al. 2022).

#### 2.2.2 Carcass

The carcass traits considered were ribeye area (REA, cm^2^) and rump fat thickness (RFT, mm); these phenotypes were obtained through measurements collected in vivo on live animals. To obtain carcass phenotypes, ultrasound images were taken of the *Longissimus dorsi* muscle between the 12th and 13th ribs (REA) and in the rump region, between the ilium and ischium at the intersection of the *Gluteus medius* and *Biceps femoris muscles* (RFT), using the ALOKA 500 V equipment with a 3.5 MHz linear probe.

#### 2.2.3 Fertility

The age at first calving (AFC, months), probability of early calving (PPC30, %), stayability (STAY, %), accumulative cow productivity (ACP, kg weaned calf/cow/year), age at puberty for males (APM), and scrotal circumference at 365 days of age (SC365) were considered in this study. All heifers that underwent the sexual precocity program were exposed to reproduction during the weaning year as part of the early calving probability (PPC30) determination process. Those females that confirmed pregnancy and gave birth to a live calf by 30 months of age received a score of 2 (success), while the others that failed received a score of 1 (failure). For stayability (STAY), cows that had at least three calvings by 76 months of age received a score of 2 (success), while those that did not meet the previous criterion received a score of 1 (failure).

The age at first calving (AFC) was defined as the age, in months, of the heifer at her first calving. Annual cow productivity (ACP) was calculated based on the average weight of weaned calves over time, considering sexual precocity, maternal ability, and the cow's reproductive regularity. The animals underwent testicular ultrasonography and andrological clinical examination to determine the males' puberty age. According to the assessment, the animals were categorised as super‐early (pubertal at ≤ 14 months of age), early (puberty between 14 and 17 months of age), or traditional (puberty > 17 months of age) (Silva Neto et al. 2020). Scrotal circumference at 365 days of age was adjusted by age.

#### 2.2.4 Feed Efficiency

Feed efficiency traits were obtained using the Intergado and GrowSafe electronic systems, which monitor individual feed intake based on the animals' visits to the feed bunks. Feed efficiency tests followed the guidelines established by Mendes et al. 2020, for assessing individual feed intake in beef cattle using both electronic systems. Animals were kept in collective or individual pens and subjected to a 21‐day adaptation period followed by a valid 70‐day testing phase. Throughout this period, each animal's average weight was recorded via manual weighing every 14 days or through automated weighing platforms (Intergado).

To obtain residual feed intake (RFI, kg of dry matter/day), the average daily gain (ADG) (kg/day) and metabolic live weight $\left({\mathrm{MW}}^{0.75}\right)$ were calculated. ADG was estimated using the linear regression coefficient of weight concerning the days in the test for the animals (DET) using the lm function in the R program with the following equation (Koch et al. 1963):

$$y_{i}=\alpha +\beta *{\mathrm{DET}}_{i}+\varepsilon_{i}$$

where: $y_{i}$ represents the weight of the animal; α is the intercept of the regression equation representing the initial weight; β is the linear regression coefficient representing ADG; ${\mathrm{DET}}_{i}$ represents the day in the test for the nth observation; e $\varepsilon_{i}$ is the error associated with each observation.

Considering live weight, the metabolic weight (${\mathrm{MW}}^{0.75}$), was calculated using the formula below (Koch et al. 1963):

$${\mathrm{MW}}^{0.75}={\left[\alpha +\beta *\left(\frac{{\mathrm{DET}}_{j}}{2}\right)\right]}^{0.75}$$

where: α represents the live weight at the beginning of the feed efficiency test; β stands for average daily weight gain, and ${\mathrm{DET}}_{j}$ represents the days in test.

Daily dry matter intake (DMI, kg/day) was derived from the mean of all valid individual daily intake values electronically recorded by the Intergado and GrowSafe systems during the test period.

Residual feed intake (RFI) was calculated as the difference between predicted and observed dry matter intake, using a regression equation based on live weight (${\mathrm{MW}}^{0.75}$), and average daily weight gain (ADG), following the methodology proposed by Koch et al. (1963).

*Y* = *β*0 + *β*1 (ADG) + *β*2 (${\mathrm{MW}}^{0.75}$) + *ε* (RFI) where: *Y* individual feed intake; *β*0 intercept; *β*1 partial regression coefficient of daily dry matter intake on average daily weight gain; *β*2 partial regression coefficient of dry matter intake on live weight; and ε: residual error of the regression, i.e., residual feed intake.

Residual live weight gain (RG) was calculated as the difference between the observed ADG and the estimated based on MW^0.75^ and DMI (Koch et al. 1963; Berry and Crowley 2012).

*Y* = *β*0 + *β*1 (DMI) + *β*2 (${\mathrm{MW}}^{0.75}$) + *ε* (RG) where *β*_*0*_ is the intercept, *β*_*1*_, and *β*_*2*_ are the regression coefficients of DMI and ${\mathrm{MW}}^{0.75}$, respectively; and ε is the residual error, i.e., RG.

#### 2.2.5 Body Composition

The calculation for the frame score was pesrformed based on the equation developed by Guimarães (2020), using the method of multiple linear regression prediction applying different equations for males (1) and females (2):

(1) $$\begin{array}{cc} \text{FRAME MALES}= & -20.35+0.1305\times \mathrm{REA}+0.2633\times \mathrm{BFT}\\ & -0.5901\times \mathrm{RFT}+0.1139\times \mathrm{HH}+0.0056\times \mathrm{AGE} \end{array}$$

(2) $$\begin{array}{cc} \text{FRAME FEMALES}= & -11.87+0.1316\times \;\mathrm{REA}-0.2457\times \mathrm{BFT}\\ & -0.6218\times \mathrm{RFT}+0.1139\times \mathrm{HH}\\ & +0.0009507\times \mathrm{AGE} \end{array}$$

where: REA, BFT, RFT, HH and AGE are ribeye area (cm^2^), subcutaneous backfat thickness (cm), rump fat thickness (cm), hip height (cm) and age (days) at ultrasound measurement, respectively.

#### 2.2.6 Novel Phenotypes

Novel phenotypes are characterised as traits not routinely included in traditional genetic evaluations, yet they present potential to improve both production efficiency and economic returns. The novel phenotypes analysed were accumulated feedlot profitability (AFP) and feedlot profit per kilogram of liveweight gain (PFT). @Tech's algorithms are designed to make full use of this data and can be collected for up to 150 days, longer than the standard 80‐day data collection period in our study. The BeefTrader Decision Support System (Albertini et al. 2017) generates the profitability phenotypes used by the commercial tool Livestock Profit Tool (LPT) to identify the most profitable animals in the herd. The system uses animal growth modelling to define the best day for animals to leave the feedlot.

The BeefTrader algorithm uses animal traits as input variables (gender, breed, body condition score, age, initial weight, initial date, among other exogenous factors that impact growth dynamics), daily weights individually collected through a weighing sensor (daily basis), and information on the nutritional composition of the diets (Albertini et al. 2017; Biase et al. 2022). The records of the DMI for obtaining the new phenotypes were collected from animals participating in feed efficiency trials, following the same guidelines as (Mendes et al. 2020), as mentioned in the section on feed efficiency traits. Based on this information, adjusted for local conditions, weight prediction was carried out in two steps: based on the biology of each animal and with the nutritional data and animal daily weight profile (observed or predicted), it is possible to estimate an optimal growth function for the animals (Step 1); from there, a dynamically adjusted linear or non‐linear regression is performed using the least squares method on the weights to fit the predicted growth curve (Step 2).

From the predicted growth curve, it is possible to find other variables required by the model, including animal performance in terms of growth and composition of gain, as well as economic and environmental factors (Gionbelli et al. 2016; Albertini et al. 2017). To assess the profit obtained by meat producers, it is common to use the unit of measurement ‘arroba’ in Brazil. In this study, the ‘arroba’ unit is defined as equivalent to 15 kg, following the standard practice in the national livestock industry. Therefore, for the purposes of this study, the term ‘arroba’ in this equation will be used to represent the profit obtained for each 15 kg of meat produced. The equations for calculating phenotypes are presented below:

Accumulated arroba:

(3) $$\left(\mathrm{sbw}*\mathrm{cdf}/100\right)/15$$

where sbw: Shrunk Body Weight (kg)—96% of the Body Weight. cdf: Carcass Dressing (%).

The carcass dressing used was 55.34% for females and 58.55% for entire males. These are real values from the company's customer database and are close to those found in the literature, which range from 54.9% to 60.6% (Arcanjo et al. 2024; Anaruma et al. 2020; Rezende et al. 2019; Rodrigues et al. 2003; Moreira et al. 2003).

Arroba* Gain

(4) $$\text{accumulated kilograms}=\text{accumulated arroba}\;\left[d\right]-\text{initial gain}\;\left[1\right]$$

where accumulate arroba = accumulated $\text{kilograms}\;\left(\text{each}\;15\mathrm{kg}\right)$ on a specific day. [d] = final day of the period to be considered, in this case 80. [1] = first day of the feedlot.

Daily Cost

(5) $$\mathrm{DMI}*\text{diet price}\;\left(\mathrm{kg}\right)+\text{feedlot daily overhead}$$

where: DMI = Dry Matter Intake (kg). diet_price_kg = diet cost ($/kg). feedlot_daily_overhead = non‐feed cost ($).

Daily Revenue

(6) arrobaprice*arrobagain

where: arroba_price = price of the arroba ($/15 kilograms). arroba gain = (referrig to each15kilograms/day).

Daily Profit

(7) revenue daily−cost daily

where: revenue daily = daily revenue ($). Cost daily = daily cost ($). Total revenue, cost and profit

(8) $$\sum_{\text{time}}\text{daily revenue}$$

(9) $$\sum_{\text{time}}\text{daily cost}$$

(10) $$\sum_{\text{time}}\text{daily profit}$$

where: daily revenue = see section 4. daily cost = see section 3. daily profit = see section 5.

Cost and Profit per kilograms*

$$\text{total cost}/\text{arroba}\;{\text{gain}}_{t}$$

$$\text{total profit}/\text{arroba}\;\;{\text{gain}}_{t}$$

where: Total profit = see section 8. Total cost = see section 7. ${\text{arroba gain}}_{t}$ = each15kilograms gain over time, see section 2.

### 2.3 Standardisation of Costs and Arroba Pricing

#### 2.3.1 Food Cost

Even considering the effect of the batch (animals evaluated by farm) in the analyses, all common foods between batches, especially among farms, had their prices standardised to set up the food cost (e.g., for corn silage, the price was always the same for the different lots, and so for all common foods in the diet). Based on the cost of natural matter (as feed) and the percentage of dry matter (DM), from the measurement of each animal's daily individual intake, the food cost for everyone was imputed over the 80‐day evaluation period. It is important to note that after the adaptation period, there were 80 days of data collection on weight, DM intake (DMI), and food and non‐food costs (operational cost), all individual, to obtain the measure of accumulated profit and profitability per arroba* gained by the evaluated animal.

#### 2.3.2 Non‐Food Cost (Operational Cost)

The non‐feed cost was also set at the same value for all evaluated batches with the aim of standardising this cost source in the process, and it's a source that doesn't affect the animals' performance.

#### 2.3.3 Price Paid Per Kilograms (arroba*)

The arroba price for all batches was standardized to the prices at the time of data collection, with the aim of ensuring that the revenue per arroba was equal for all animals. The prices followed those indicated by Center for Advanced Studies in Applied Economics (CEPEA, https://www.cepea.esalq.usp.br/br/indicador/boi‐gordo.aspx)**—University of São Paulo (USP).

*accumulated feedlot profitability (AFP): accumulated feedlot profitability in monetary units in the 80‐day period of feedlot; **profit per kilogram of liveweight gain (PFT): profitability per 15 kg of weight gained in feedlot.

### 2.4 Data Editing and Statistical Analysis

The contemporary groups (CG) for profitability, growth, fertility, carcass, and feed efficiency traits were formed by farm, management lot, sex, year, and birth season (dry season: April–September and rainy season: October–March). For the profitability and feed efficiency traits, the identification of the feed efficiency test was also considered to form the CG. Animals belonging to CG with fewer than four individuals, as well as those without identification of sire and dam or those lacking phenotypic records within ±3.5 standard deviations from the meaning of the CG, were excluded from the analyses. The number of records and the descriptive statistics for the traits studied are presented in Table 1.

**TABLE 1:** Descriptive statistics and number of animals with phenotypic records (N) and contemporary groups (NCG) for growth, reproduction, carcass, feed efficiency, and profitability‐related traits in Nelore cattle.

TRAIT (unit)	*N*	MEAN	SD	MIN	MAX	CV	NCG
AFP ($)	3614	151.84	66.45	−17.98	420.06	43.76	226
PFT ($/kg)	3614	36.21	9.75	−13.33	51.23	26.93	226
W450 (kg)	55.952	289.97	62.70	119.00	592.00	21.62	2181
DMI (kg/day)	9910	8.06	1.87	3.18	18.74	23.21	219
RFI (kg of DMI/day)	9910	0.00	0.67	−4.93	4.69	—	219
RG (kg of ADG/day)	11.125	0.00	0.28	−1.07	1.06	—	251
REA (cm^2^)	36.170	57.20	12.70	20.45	116.35	22.21	1496
RFT (mm)	36.115	4.28	2.70	0.13	24.39	63.24	1496
FRAME	10.567	5.36	2.16	−6.34	15.58	40.38	425
PPC30 (%)	7116	1.48	0.50	1.00	2.00	33.60	126
AFC (month)	27.457	35.26	7.17	21.00	49.00	20.33	463
ACP (kg/cow/year)	14.983	140.86	33.34	45.00	331.00	23.67	286
STAY (%)	18.940	1.34	0.48	1.00	2.00	35.34	276
SC365 (cm)	19.300	21.46	2.97	12.90	34.70	13.84	2303
APM (months)	3422	16.62	3.89	8.73	22.00	23.45	32

Abbreviations: ACP, accumulated cow productivity; AFC, age first calving; AFP, accumulated profitability; APM, age at puberty in males; DMI, dry‐matter intake; FRAME, frame score; PFT, profit per kilogram of liveweight gain; PPC30, probability of precocious calving at 30 months of age; REA, rib eye area; RFI, residual feed intake; RFT, rump fat thickness; RG, residual live weight gain; SC365, scrotal circumference at 365 days of age; STAY, stayability; W450, weight at 450 days of age.

### 2.5 Variance Components and Genetic Parameters Estimation

The (co)variance components and genetic parameters were estimated using a multiple‐trait animal model through Bayesian inference. This approach employed the Gibbs sampling algorithm implemented in the GIBBSF90+ software (Misztal et al. 2014) for both linear and categorical traits. Two groups of mult‐trait models were constructed, as follows: (1) Model 1 (*n* = 9) considered the traits AFP, PFT, and W450, DMI, RFI, RG, REA, RFT and FRAME; (2) Model 2 (*n* = 8), the traits AFP, PFT, and reproductive traits PPC30, STAY, AFC, ACP APM and SC365 were considered. The general animal model used was:

y=Xβ+Za+e.

where y is the vector of observations; β is the vector of fixed effects (CG); a is the vector of direct additive genetic effects, and e is the vector of random residual effects associated with the observations. X and Z are incidence matrices relating b and a to y. The assumptions made in the model were: $E\left[y\right]$ $=\mathrm{X\beta};\mathrm{Var}\left(a\right)A⨂G\;\text{and}\;\mathrm{Var}\left(e\right)=I⨂R,$ where *G* is the direct genetic (co)variance effects; *A is the* relationship matrix; *I* is the identity matrix; *R* is the residual (co)variance matrix, and ⨁ is the direct product of matrices. The general structure of the variance and covariance matrices of the random effects in the models was:

$$\left[\begin{array}{c} a\\ e \end{array}\right]\sim N\;\left(0,V\right);V=\left[\begin{array}{c} G⨂H\;0\;\\ \;0\;R⨂I \end{array}\right]$$

where G is the (co)variance matrix of the direct additive genetic effects, H is the is a combined matrix from *A* (pedigree relationship matrix) and *G* (genomic relationship matrix); R is the residual (co)variance matrix, I is the identity matrix, and ⨁ is the direct product of the matrices.

As the multi‐trait model comprised both categorical and linear traits, it was assumed that the initial distributions of the genetic and residual random effects followed a multivariate normal distribution, according to the Bayesian approach, as described below (adapted from Bonamy et al. 2019):

$$\begin{array}{c} p\;\left(\left[\begin{array}{c} \alpha_{1}\\ \vdots \\ \alpha_{n} \end{array}\right]\;|G\right)\sim N=\left(\left[\begin{array}{c} 0\\ \vdots \\ 0 \end{array}\right],G=G_{0}⨂H\right),\\ P\left(\left[\begin{array}{c} e_{1}\\ \vdots \\ e_{n} \end{array}\right]\;|R\right)\sim N\left(\left[\begin{array}{c} 0\\ \vdots \\ 0 \end{array}\right],R=R_{0}⨂I\right) \end{array}$$

where: $G_{0}$ is the genetic (co)variance matrix; $R_{0}$ is the residual variance matrix; ⊗ direct product; *H* is the is a combined matrix from *A* (pedigree relationship matrix) and G (genomic relationship matrix); *I* is the identity matrix. The inverse of the *H* matrix ($H^{-1}$) was constructed according to Aguilar et al. (2010) as:

$$H^{-1}=A^{-1}+\left[\begin{array}{cc} 0 & 0\\ 0 & G^{-1}-A_{22}^{-1} \end{array}\right]$$

where $G^{-1}$ is the inverse of the genomic relationship matrix and $A_{22}^{-1}$ is the inverse of numerator relationship matrix. The genomic relationship matrix (*G*) is the submatrix of the direct additive genetic and was built as per VanRaden (2008), in which $G=ZZ^{\prime }/2\sum \;p_{k}q_{k}$ with p and q being the allele frequencies of marker K.

In the Gibbs Sampling implementation, 600,000 iterations were employed, with an initial burn‐in of 100,000 iterations and a sampling interval of 100 iterations (thin). Convergence of the Monte Carlo Markov Chain (MCMC) was assessed using the Geweke Test (Geweke 1992), available in the R package Bayesian Output Analysis Program (BOA) (Smith 2005), as well as through visual evaluation using trace‐plot graphs.

The estimated heritability coefficients were categorized following the guidelines established by Bourdon (1997), classifying them as low (below 0.20), moderate (ranging from 0.20 to 0.40), and high (above 0.40). For genetic and phenotypic correlations, the recommendation of Hill (2013) was adhered to, considering them as low (less than 0.30), moderate (ranging from 0.30 to 0.70), and high (greater than 0.70).

## 3 Results and Discussion

The variance components and heritability estimate for accumulated profitability, Profit per kilogram of liveweight gain, growth, carcass, reproduction, and feed efficiency are presented in Table 2. The heritability estimates were moderate to low (0.18), suggesting that genetic progress can be achieved through selection for AFP. The estimated heritability for PFT was low (0.02). PFT is an economic trait that reflects the relationship between two components: the economic, made up of the financial return per kg of meat produced, and the biological, which includes different traits such as carcass yield and weight. In other words, it is directly affected by a complex action between economic and biological components. Thus, the partitioning of variance components or the estimation of the fraction of additive genetic components that are responsible for the expression of the phenotype becomes difficult for traits resulting from a relationship of more traits.

**TABLE 2:** Posterior mean and high probability density (HPD^a^) for direct additive genetic ($\sigma_{a}^{2}$), residual ($\sigma_{e}^{2}$) variances and heritability (SD, standard deviation) for growth, carcass, feed efficiency, reproductive and feedlot profitability related traits in Nelore cattle.

Trait	$\sigma_{a}^{2}$	$\sigma_{e}^{2}$	*h* ^2^	±SD	HPD
AFP	6321.23	28687.29	0.18	0.04	0.095 to 0.255
PFT	10.42	448.46	0.02	0.00	0.020 to 0.026
W450	292.87	539.77	0.35	0.01	0.326 to 0.375
DMI	0.23	0.62	0.27	0.03	0.231 to 0.316
RFI	0.07	0.41	0.15	0.02	0.113 to 0.185
RG	0.0004	0.03	0.12	0.02	0.085 to 0.165
REA	11.81	26.02	0.31	0.01	0.287 to 0.339
RFT	0.52	1.13	0.31	0.01	0.292 to 0.337
FRAME	0.55	1.17	0.32	0.02	0.291 to 0.351
PPC30	0.46	1.00	0.31	0.04	0.225 to 0.397
STAY	0.20	1.00	0.16	0.02	0.128 to 0.210
AFC	2.36	20.77	0.10	0.01	0.078 to 0.125
ACP	162.28	514.36	0.24	0.02	0.195 to 0.283
SC365	1.15	1.96	0.36	0.02	0.322 to 0.413
APM	5.79	7.51	0.43	0.05	0.326 to 0.535

Abbreviations: ACP, accumulated cow productivity; AFC, age first calving; AFP, accumulated profitability; APM, age at puberty in males; DMI, dry‐matter intake; FRAME, frame score; PFT, profit per kilogram of liveweight gain; PPC30, probability of precocious calving at 30 months of age; REA, rib eye area; RFI, residual feed intake; RFT, rump fat thickness; RG, residual live weight gain; SC365, scrotal circumference at 365 days of age; STAY, stayability; W450, weight at 450 days of age.

^a^ Confidence intervals at 95%.

To date, there have been no studies on estimating genetic parameters for profitability‐related traits measured directly in cattle confinement. However, with the development of new phenotyping technologies, it is possible to identify and quantify new traits and potential environmental factors that influence phenotypic variation (Berry 2023). This novel perspective underscores the importance of considering new traits, in addition to conventional ones, in the selection process, demonstrating the ongoing evolution of research and industry efforts toward improved economic indicators and sustainability.

The estimated heritability for W450 was moderate (0.35), in agreement with findings in Nelore cattle, as reported by Bonamy et al. 2019, Negreiros et al. 2022 and Silva Neto et al. 2023, ranging from 0.26 to 0.37. The heritability estimates for SC365 and APM were moderate to high, 0.36 and 0.43, respectively, and similar to those reported by Silva Neto et al. 2020, which were 0.33 and 0.30, respectively. The PPC30 heritability estimates were moderate, 0.28, and similar to those reported by Bonamy et al. 2019, and Negreiros et al. 2022, in Nelore animals, which values were 0.29 and 0.28, respectively.

The heritability estimates for STAY in Nellore females reported in the literature ranged from 0.09 to 0.16 (Van Melis et al. 2010; Bonamy et al. 2019; Costa et al. 2020), closely resembling the one obtained in this study (0.16). For AFC and ACP, the estimated heritability estimates were low to moderate (0.10 and 0.24, respectively; Table 2), indicating a considerable environmental influence on these traits. Similar heritability values for AFC and ACP were also reported in Nelore animals by Costa et al. 2020, Van Melis et al. 2010, and Kluska et al. 2018, ranging from 0.08 to 0.16.

The heritability estimates for REA (0.31) and RFT (0.31) suggest that these traits have a rapid response to selection. These results are in line with previous studies (Silva Neto et al. 2023; Londoño‐Gil et al. 2022), whose values ranged from 0.28 to 0.33. Regarding feed efficiency traits, the heritability estimate for RFI (0.15) was low and lower than that reported by Kava et al. 2023 and Gomes et al. 2023, both in Nelore cattle (0.55 and 0.37, respectively). For DMI, the heritability estimate was moderate (0.27), similar to estimates in the literature ranging from 0.29 to 0.45 in Nelore cattle (Kava et al. 2023; Gomes et al. 2023; Ceacero et al. 2016). The heritability estimate for RG was 0.12, lower than the value reported by Berry and Crowley (2012) (0.28).

The genetic correlations between AFP, PFT and growth, reproduction, carcass, and feed efficiency–related traits are presented in Table 3. The estimated genetic correlations between AFP and PFT with W450 were favorable and moderate to high, 0.51 and 0.64, respectively. These results suggest that selecting animals with higher W450 would also increase AFP and PFT. Performance traits measured post‐weaning are often used in breeding programs as selection criteria (Pinheiro et al. 2012), due to their favorable genetic correlation with live weight gain efficiency and final carcass weight at slaughter (Silva et al. 2018; Pinheiro et al. 2012).

**TABLE 3:** Posterior mean and high probability density (HPD^a^) for genetic correlations between traits related to feedlot profitability with growth, reproduction, carcass, and feed efficiency in Nelore cattle.

Trait	Mean	± SD	HPD
*Genetic correlation with accumulated profitability*			
W450	0.65	0.08	0.512 to 0.812
DMI	0.72	0.09	0.546 to 0.866
RFI	0.28	0.15	−0.005 to 0.544
RG	0.83	0.08	0.727 to 0.919
REA	0.43	0.09	0.263 to 0.620
RFT	−0.10	0.07	−0.246 to 0.041
FRAME	0.44	0.08	0.284 to 0.592
PPC30	0.16	0.31	−0.438 to 0.729
STAY	0.42	0.17	0.054 to 0.737
AFC	−0.19	0.20	−0.811 to 0.188
ACP	−0.14	0.19	−0.498 to 0.216
SC365	−0.05	0.12	−0.297 to 0.168
APM	−0.21	0.18	−0.547 to 0.229
*Genetic correlation with profit per kilogram of liveweight gain*			
W450	0.64	0.05	0.549 to 0.734
DMI	0.26	0.08	0.116 to 0.405
RFI	−0.23	0.08	−0.373 to −0.067
RG	0.88	0.08	0.732 to 0.969
REA	0.44	0.07	−0.329 to 0.584
RFT	−0.68	0.04	−0.757 to −0.596
FRAME	0.77	0.44	0.692 to 0.869
PPC30	0.73	0.14	0.436 to 0.926
STAY	0.76	0.19	0.352 to 0.968
AFC	−0.47	0.08	−0.621 to −0.304
ACP	−0.01	0.09	−0.208 to 0.178
SC365	−0.06	0.08	−0.210 to 0.095
APM	−0.03	0.13	−0.294 to 0.219

Abbreviations: ACP, accumulated cow productivity; AFC, age first calving; APM, age at puberty in males; DMI, dry‐matter intake; FRAME, frame score; PPC30, probability of precocious calving at 30 months of age; REA, rib eye area; RFI, residual feed intake; RFT, rump fat thickness; RG, residual live weight gain; SC365, scrotal circumference at 365 days of age; STAY, stayability; W450, weight at 450 days of age.

^a^ Confidence interval at 95%.

The genetic correlation between AFP and DMI was high (0.72) and low with PFT (0.26). These results suggest that selection for higher profitability would increase the DMI. DMI is strongly associated with feed efficiency, body weight, and growth in cattle (Herd et al. 2014; Donoghue et al. 2016). Animals that eat more dry matter usually tend to have a higher average daily gain, which results in better feed conversion (Ceacero et al. 2016). The results of these studies pointed out that genetically more profitable animals have higher feed intake and better feed efficiency.

The genetic correlation between AFP and PFT with RFI was low, 0.28 and −0.23, respectively. These results suggest that selection to improve RFI would not influence feedlot profitability. It is important to highlight that RFI is closely related to the maintenance of energy in cattle (Herd and Bishop 2000). It is an important measure to identify animals that differ in the efficiency of using energy for maintenance, as reported by Archer et al. (1999). More efficient animals, i.e., low RFI, are due to their higher metabolic efficiency, reducing the energy cost of maintenance (Tempelman and Lu 2020), which reduces demand for food without compromising the growth or size of adult animals (Koch et al. 1963). Furthermore, many studies have reported the low genetic association of RFI with output traits, such as reproduction (Bonamy et al. 2019), −0.09 to 0.08, growth, and carcass, −0.23 to 0.23 (Ceacero et al. 2016). RFI is more related to metabolizable energy efficiency than direct results in the production system, such as weight, weight gain, and rib eye area, which directly influences the profitability potential of the animals, which corroborates the low genetic correlation between RFI, and profitability obtained in this study.

The genetic correlation between the profitability indicator traits, AFP and PFT, and RG was high, 0.83 and 0.88, respectively. RG is a metric that evaluates the growth efficiency of animals, identifying those with faster growth rates than expected. These genetic correlations can be explained by the fact that animals with greater residual weight gain tend to convert feed into body weight more efficiently in feedlots, which is economically advantageous. This genetic efficiency may be related to traits such as more efficient metabolism and better feed conversion.

Moderate and favourable genetic correlation estimates between AFP and PFT with REA (0.43 and 0.44, respectively) were obtained, suggesting that selection to increase REA would likely result in higher feedlot profitability. The favourable genetic correlation between profitability and REA can be a relevant parameter for selection in production systems during the rearing and finishing phases. The REA is associated with the degree of muscling, edible mass, and yield of high‐value meat cuts (Malheiros et al. 2020). This suggests that animals with greater muscularity, reflected by the ribeye area, can produce a larger quantity of higher‐quality meat. As a result, these animals are generally more valued in the industry and generate higher profits per animal. Therefore, by focusing on the selection of animals with a higher ribeye area, producers can expect an increase in the profitability of beef production, as these traits are positively correlated.

The genetic correlations between AFP and PFT with RFT were low and moderate, −0.10 and −0.68, respectively, implying that selection for a higher degree of carcass fatness would not lead to more profitable animals in the feedlot. To obtain animals with greater AFP, PFT, and finishing, these traits must be selected simultaneously in genetic improvement programs. Subcutaneous fat thickness is essential in determining meat product quality, as it shields the carcass from fibre shortening during the cooling process (Malheiros et al. 2015).

The low to high genetic correlation (0.16 and 0.73, respectively) obtained between AFP and PFT with the female sexual precocity indicator trait (PPC30) indicates that selection to improve PPC30 would also improve feedlot profitability. These indicate that early females have a better economic return and productivity, due to the herd's better reproductive efficiency, especially in commercial herds.

The estimated genetic correlation between AFP and PFT and stayability was moderate to high, at 0.42 and 0.76, respectively, suggesting a favourable association between these traits. Stayability is the female's ability to remain in the herd until a certain age (Van der Westhuizen et al. 2001; Maiwashe et al. 2009). The genetic correlation obtained in this study suggests that a female's profitability intricately links to her ability to remain in the herd and produce more kilograms of weaned calves throughout her life. Furthermore, the production of kilograms of calves is directly associated with the cow's reproductive efficiency, whose factors include age at first calving, conception rate, calving interval, and milk production capacity.

Similarly, the genetic correlation estimates between AFP and PFT with AFC were moderate to low, −0.19 and −0.47, respectively. Therefore, the negative but favorable correlations indicate that reducing the age at first calving may be associated with higher AFP and PFT. Although sexual precocity and age at first calving are not directly associated with meat production in the finishing phase, the genetic merit of females can indirectly influence the quality and quantity of calves produced, contributing to the availability of animals for finishing. On the other hand, genetic correlation estimates between ACP, SC365, APM, AFP, and PFT were close to zero, ranging from −0.01 to −0.21 (Table 3), indicating that selection to increase scrotal circumference, such as reducing the age at puberty in males, or cow accumulated productivity would not increase or affect feedlot profitability.

The genetic correlation estimates between AFP and PFT with FRAME (0.44 and 0.77, respectively) indicate a moderate to high and favourable association. Therefore, selecting FRAME would increase the feedlot profitability. Animals with a balanced body structure, that is, medium‐sized, tend to convert feed into weight gain more efficiently, with adequate fat deposition. This contributes to quicker readiness for slaughter. Breeders can use this information to select animals better suited to the specific conditions of the environment and management conditions in which they will be raised, which can lead to higher productivity and, consequently, increased profit per unit of weight.

The residual correlations between AFP, PFT and growth, reproductive, feed efficiency, and carcass traits are described in Table 4. In general, residual correlations for most traits with AFP, and PFT were low and close to zero, suggesting a low association between traits relative to non‐additive components (Falconer and Mackay 1996). This suggests that shared environmental factors and non‐additive genetic effects do not strongly influence the traits under study. However, except for the moderate residual correlation between profitability and W450 and DMI (0.21 and 0.21, respectively), it is indicated that variations similarly influence these traits in environmental conditions and favor these traits in the same direction.

**TABLE 4:** Posterior mean and high probability density (HPD) for residual correlations between feedlot profitability‐related traits with growth, reproduction, carcass, and feed efficiency in Nelore cattle.

Trait	Mean	±SD	HPD
*Residual correlation with accumulated profitability*			
W450	0.21	0.03	0.146 to 0.276
DMI	0.21	0.03	0.150 to 0.262
RFI	−0.05	0.03	−0.098 to 0.011
RG	0.57	0.02	0.539 to 0.603
REA	0.12	0.03	0.063 to 0.170
RFT	0.13	0.03	0.073 to 0.177
FRAME	0.03	0.04	−0.0.53 to 0.107
PPC30	0.16	0.18	−0.198 to 0.522
STAY	0.76	0.05	0.665 to 0.869
AFC	0.02	0.07	−0.121 to 0.157
ACP	0.17	0.33	−0.397 to 0.764
SC365	0.18	0.05	0.083 to 0.282
APM	0.07	0.08	−0.101 to 0.235
*Residual correlation with profit per kilogram of liveweight gain*			
W450	0.09	0.03	0.029 to 0.161
DMI	0.06	0.02	0.014 to 0.104
RFI	0.07	0.21	0.032 to 0.115
RG	0.50	0.02	0.474 to 0.536
REA	0.05	0.02	0.008 to 0.097
RFT	0.07	0.03	0.029 to 0.112
FRAME	0.01	0.03	−0.442 to 0.066
PPC30	0.34	0.23	−0.183 to 0.685
STAY	0.90	0.05	0.860 to 0.958
AFC	0.02	0.07	−0.126 to 0.159
ACP	0.11	0.35	−0.480 to 0.733
SC365	0.15	0.05	0.015 to 0.206
APM	0.01	0.08	−0.135 to 0.164

Abbreviations: ACP, accumulated cow productivity; AFC, age first calving; APM, age at puberty in males; DMI, dry‐matter intake; FRAME, frame score; PPC30, probability of precocious calving at 30 months of age; REA, rib eye area; RFI, residual feed intake; RFT, rump fat thickness; RG, residual live weight gain; SC365, scrotal circumference at 365 days of age; STAY, stayability; W450, weight at 450 days of age.

Based on the results, these two new novel traits (AFP and PFT) can be used as support tools for efficiently managing feedlot operators' daily operations and as selection criteria for intensive beef cattle systems. Applying AFP and PFT in commercial herds allows us to classify the animals that would have a lower return than expected from those with higher genetic potential for profitability The animals with higher genetic potential for feedlot profitability are genetically associated with greater weight gain, carcass muscle deposition, and carcass yield. These animals, which require shorter feedlot duration lengths, can be identified early, and allocated differently from animals with lower returns, enabling precise and more efficient management of resources.

For breeding herds, the genetic evaluation of genetic profit can help producers make decisions about the economic return based on their selection decisions (Hassanvand‐Javanmard et al. 2016) and the prediction of genetic matings, when these traits are used. This approach contributes to optimising business operations while reducing environmental impacts.

## 4 Conclusion

The results obtained in this study showed that AFP and PFT have genetic variability and can respond to selection, although the response to selection for PFT is slow. The moderate to high and favourable genetic correlations revealed that W450, REA, FRAME, and DMI are the traits most associated with feedlot profitability, while W450, FRAME, and REA were the most relevant for PFT. The results showed how much fertility and precocity traits affect the economic performance of animals in finishing due to the moderate to high and favourable genetic correlations with AFP and PFT. In other words, the profitability of animals in fattening can also be affected by the quality of the females.

Therefore, these traits can be included as additional tools in the selection criteria, since these genetic indicators make it possible to classify animals based on the genetic evaluation of profitability in feedlots, which are important for management and decision‐making.

## Author Contributions

**Letícia Pereira:** conceptualization, methodology, formal analysis, writing – original draft preparation. **Fernando Baldi:** conceptualization, methodology, formal analysis, writing – original draft preparation. **Guilherme Jordão Magalhães Rosa:** methodology, formal analysis, writing and review. **José Bento Sterman Ferraz:** conceptualization. **Tiago Zanett Albertini:** data curation, writing – review, and editing. **Minos Carvalho:** data curation, writing – review, and editing. **Raysildo Barbosa Lobo:** data curation, review. **Eduardo da Costa Eifert:** conceptualization, writing and review. **Elisa Peripolli:** conceptualization. **Cláudio Ulhôa Magnabosco:** conceptualization, methodology, formal analysis, writing and review.

## Conflicts of Interest

The authors declare no conflicts of interest.

## Data Availability

The data that support the findings of this study are available on request from the corresponding author. The data are not publicly available due to privacy or ethical restrictions.
